# A Fully Printable Strain Sensor Enabling Highly‐Sensitive Wireless Near‐Field Interrogation

**DOI:** 10.1002/advs.202411346

**Published:** 2025-01-21

**Authors:** Hassan A. Mahmoud, Hussein Nesser, Tarek M. Mostafa, Shehab Ahmed, G. Lubineau

**Affiliations:** ^1^ Mechanical Engineering Program Physical Science and Engineering Division King Abdullah University of Science and Technology (KAUST) Thuwal 23955‐6900 Kingdom of Saudi Arabia; ^2^ Mechanics of Composites For Energy and Mobility Lab King Abdullah University of Science and Technology (KAUST) Thuwal 23955 Saudi Arabia; ^3^ CEMSE Division King Abdullah University of Science and Technology Thuwal 23955 Saudi Arabia

**Keywords:** capacitive strain sensors, embedded sensors, NFC, passive RF sensors, SHM

## Abstract

Wireless, passive, and flexible strain sensors can transform structural health monitoring across various applications by eliminating the need for wired connections and active power sources. Such sensors offer the dual benefits of operational simplicity and high‐function adaptability. Herein, a novel wireless sensor is fabricated using radio frequency (RF) technology for passive, wireless measurement of mechanical strains. Previously introduced concept of coupling piezoresistive electrodes is utilized with capacitive sensors to ensure high‐sensitivity capacitive sensing. For the first time, it is implemented and demonstrated here as a fully printable, inexpensive, and ready‐to‐use device utilizing the recent advances in piezoresistive inks and screen‐printing technologies. The near‐field communication (NFC) tag features an inductor ‐ capacitor (LC) resonant circuit with a distinct resonant frequency. The sensor exhibits high sensitivity and detects substantial variations in capacitance, with a gauge factor (GF) of ≈16 at 20 MHz for strain levels below 5%. Within the wireless framework, the sensor achieves a significant shift in resonance frequency (GF of ≈2.2). It also exhibited excellent performance in wirelessly monitoring the strain in a glass fiber‐reinforced polymer (GFRP) specimen during the bending test. The results confirm the potential applicability of the sensor as an embedded sensor for monitoring various types of composite structures. This confirms the potential of the sensor for use in composite structures as an embedded sensor.

## Graphical abstract

Wireless, passive, and flexible strain sensors revolutionize structural health monitoring by removing wired connections and power needs. A novel radio frequency (RF)‐based wireless sensor is presented for passive strain measurement, that integrates piezoresistive electrodes with capacitive sensors for high sensitivity. This fully printable, low‐cost device using piezoresistive inks and screen‐printing, features an Near field communication (NFC) tag with an inductor‐capacitor (LC) resonant circuit. The sensor demonstrates high sensitivity and significant shifts in the resonance frequency.

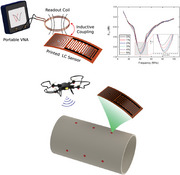

## 1 Introduction

Wireless sensors have evolved considerably in recent years, revolutionizing the way we monitor and interact with our environment. These sensors can precisely measure various physical and environmental conditions due to their compact size, high sensing accuracy, and low power consumption.^[^ ^1^ ^]^ They are now integral to a range of applications including biomedical health monitoring,^[^ ^2^, ^3^, ^4^ ^]^ structural integrity monitoring,^[^ ^5^, ^6^, ^7^ ^]^ environmental monitoring,^[^ ^8^, ^9^ ^]^ gas detection,^[^ ^10^, ^11^ ^]^ and underground applications.^[^ ^12^ ^]^ Owing to their reliability, energy efficiency, easy deployment, and minimal invasiveness. Passive wireless sensors, which can operate without an external power source, hold substantial promise due to cost‐effectiveness and non‐intrusiveness, as well as their low maintenance requirements and environmental impact.^[^ ^5^, ^13^, ^14^ ^]^ Their minimalist design facilitates integration with various structures to monitor and collect data on critical indicators of structural health without compromising structural integrity. A passive inductor‐capacitor (LC) resonant circuit is an ideal design for remote sensing because it can be powered externally without a battery, leading to a very compact sensor design.^[^ ^15^ ^]^ This design also allows fabrication on a flexible substrate, making the sensor adaptable to various surfaces. With the expansion of the Internet of Things (IoT) into wearable and implantable devices, LC circuit‐based sensors are being actively studied across various disciplines^[^ ^16^ ^]^ for measuring different parameters such as strain,^[^ ^5^, ^17^ ^]^ pressure,^[^ ^18^ ^]^ temperature,^[^ ^19^ ^]^ humidity,^[^ ^20^ ^]^ and pH.^[^ ^21^ ^]^

As wireless technology advances, the quest for more efficient and sensitive strain sensors continues. A key component in this pursuit is the LC circuit. An LC circuit typically comprises a capacitor connected to a coil to form an LC resonant circuit with a unique resonant frequency.^[^ ^22^ ^]^ In a capacitive wireless strain sensor, capacitance serves as a sensing unit, where changes in capacitance measure external stimuli. The shift in the resonant frequency of the oscillating circuit results from changes in the capacitance of the sensor.^[^ ^5^ ^]^ The change in capacitance of the classical capacitive sensors depends on the variation in the geometrical parameters of the two conductive electrodes and the dielectric layer.^[^ ^23^ ^]^ However, this reliance on changes in geometry limits the gauge factor (GF) of the sensor, particularly at low strain levels, resulting in minimal shifts in the resonant frequency.^[^ ^24^ ^]^ We previously fabricated a wireless strain sensor based on an LC resonant circuit,^[^ ^5^ ^]^ by introducing nano‐caracks to parallel metal plate capacitors, which creates a piezoresistive response that modified the electromagnetic wave penetration, achieving high sensitivity, and causing a large shift in the resonant frequency with GF of 50 for strain less than 1%. This sensor was fabricated using microfabrication techniques that are costly, time‐consuming, and not scalable. To speed up the production process and reduce costs while retaining the benefits of remote sensing and high sensitivity, we explored using planar capacitors and piezoresistive materials compatible with classical batch production technologies such as flat or roll‐to‐roll screen printing.

In this study, a novel wireless, passive sensor that uses an LC resonant circuit was fabricated using screen printing technology on a flexible polyimide (PI) substrate with conductive and piezoresistive inks. Our innovative approach combines capacitive and piezoresistive detection methods to significantly improve the sensitivity of wireless strain measurements. The high sensitivity was achieved by modulating the transmission line and altering the penetration of electromagnetic signals at various strain levels. Owing to its flexibility and sensitivity, the sensor can be easily implanted and integrated with different structures for structural health monitoring (SHM) of composite structures, buildings, bridges, aircraft, and pipelines. Moreover, the sensor can be used to detect minute deformations or stresses that could indicate potential failures, ensuring timely maintenance, and enhancing the safety of critical infrastructure.

## 2 Results and Discussion

### 2.1 Wireless Sensor Design and Fabrication

**Figure 1:**
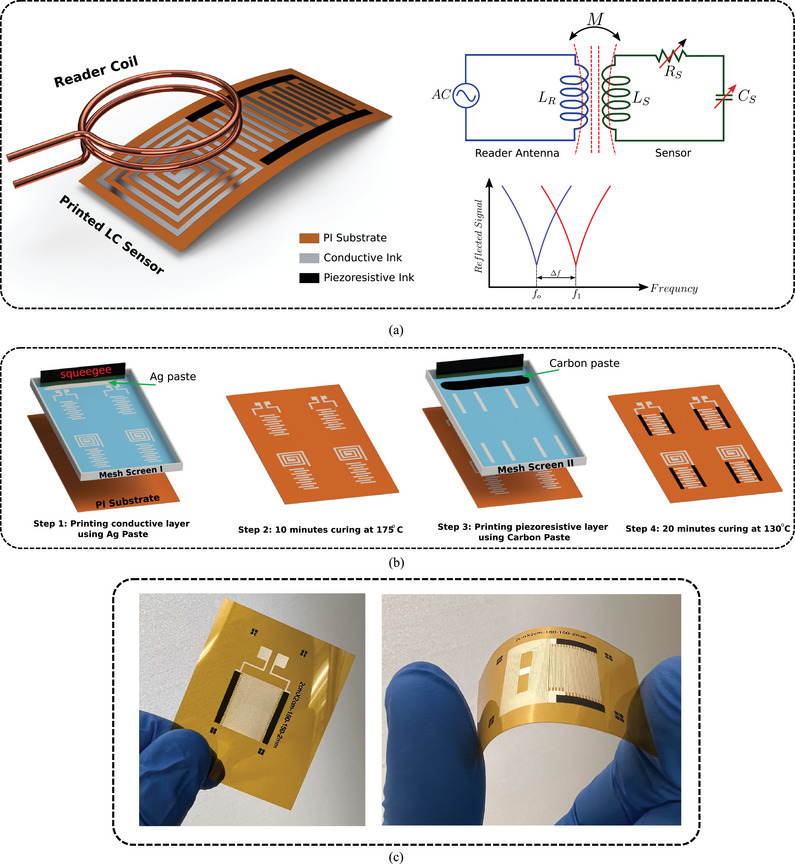
a) Schematic of the printed sensor comprising an IDEs capacitor and spiral coil forming an LC resonant circuit. The spiral coil is electromagnetically coupled with the external readout coil and any changes in capacitance from stretching result in a shift in the resonant frequency. b) Fabrication of the printed sensor using screen printing technology with two types of inks; conductive (silver paste) and piezoresistive (carbon paste). c) Optical images of the final printed IDEs capacitor and wireless LC sensor.

The wireless strain sensor (**Figure** **1**a) functioned as an LC resonant circuit with a unique resonant frequency after connecting an interdigitated electrodes (IDEs) capacitor to a planar spiral coil. An external read‐out coil is connected to the vector network analyzer (VNA) that sends and sweeps signals, including the resonant frequency of the LC circuit to create an electromagnetic coupling and resonate the system on its resonant frequency. Instead of relying on the inductive sensing technique, which has limited sensitivity compared to resistive or capacitive sensors,^[^ ^17^, ^25^ ^]^ we used the capacitor as a sensing element, and any change in the capacitor due to stretching will cause a change in the resonant frequency of the reflected signal as illustrated in Figure 1a. Classical capacitive strain sensors are guided by geometrical variations have limited sensitivity, therefore, a minimal shift is observed in the resonant frequency. This limitation restricted their range of applications, particularly in scenarios that require high sensitivity at low strain levels.^[^ ^5^ ^]^ In this work, we introduced a new sensor design by combining the piezoresistive and capacitive behavior to control the electromagnetic signal penetration depth in the capacitor of printed LC sensor, enhance the sensitivity, and achieve a remarkable shift in the resonant frequency for detecting change in the strain. Analytical analysis was performed to design the LC resonator by calculating the inductance (*L*_*S*_) and capacitance (*C*_*S*_) of the spiral coil and IDEs capacito, respectively. The IDEs capacitor was designed based on Equation S7 (Supporting Information),^[^ ^26^, ^27^ ^]^ and the inductor was designed based on the Modified wheeler formula (Equation S1, Supporting Information).^[^ ^28^ ^]^ The resonant frequency (*f*_*r*_) was calculated, as shown in Equation 1. All the equations required for designing the IDEs sensor are expressed in Equations S1– S8 (Supporting Information).

(1) $$f_{r}=\frac{1}{2\pi \sqrt{L_{S}C_{S}}}$$

### 2.2 Materials and Morphology Characterization

**Figure 2:**
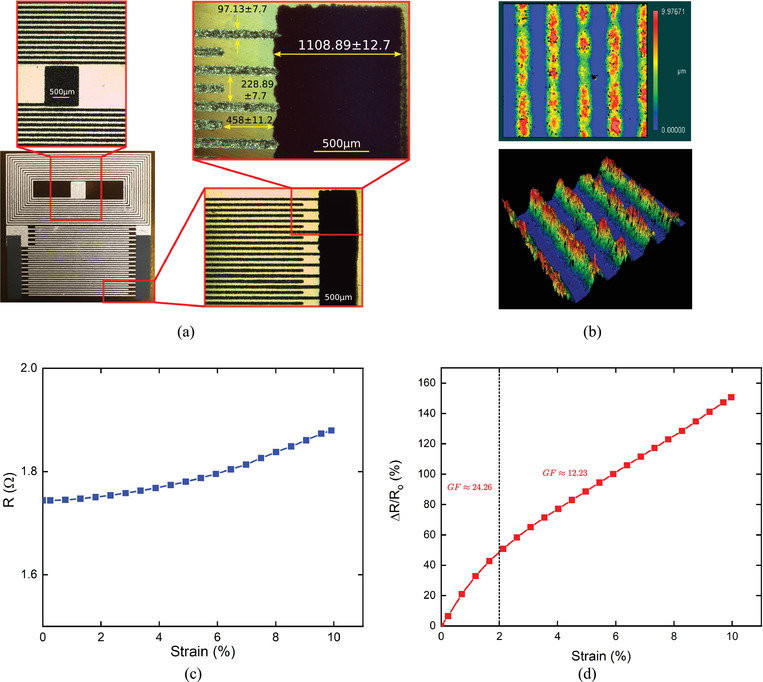
a) Optical microscopy images of the printed LC resonant circuit showing the resolution and dimensions of the printed structures. b) 3D images using an optical profilometer show the thickness distribution of the printed electrodes. c) Resistance variation in the conductive ink during stretching until 10% strain d) Relative change in electrical resistance with strain for the piezoresistive ink.

The wireless printed strain sensor was fabricated using the screen printing technology (Figure 1b) in two main stages using two mesh screen masks with different designs of IDEs capacitor and LC sensor. First, a layer of electrode fingers and coil was printed using conductive ink on a 50 µm PI substrate and cured before printing the second layer of piezoresistive electrodes. Finally, different structures of the IDEs capacitor and LC sensor were obtained and then cut into samples (Figure 1c). To close the circuit of the LC sensor, a bridge connection was created as illustrated in Figure S2 (Supporting Information). Microscopy images were captured to determine the resolution and the quality of the printed structures (**Figure** **2**a) at different scales. The fine structures of IDEs figures were printed with high resolution and perfect alignment between the conductive and piezoresistive electrodes. An optical profilometer was used to measure the 3D thickness of the printed structures. As shown in Figure 2b, the thickness of one printed layer was ≈10µm.

Using a highly conductive material for the coil in the LC sensor is vital to minimize the coil loss factor and optimize the transmitted signal to the reader coil. Therefore, highly conductive and stretchable inks were used for printing the sensor coil and IDEs fingers to maintain their high conductivity even at high strain levels. To quantify the electromechanical properties, a conductive electrode printed on a PI substrate was subjected to a uniaxial tensile test where the change in electrical resistance until 10% strain was very small and in the Ω range as presented in Figure 2c. A piezoresistive material with high resistance variation is required to enable the transmission line and control the penetration depth of the signal to achieve high sensitivity in strain detection. Figure 2d shows the variation in the relative electrical resistance of the piezoresistive ink under stretching (from 4.2 to 10.7 kΩ within 10% strain). The sensitivity of the piezoresistive ink was quantified by calculating GF using the following equation, *GF* = (Δ*R*/*R*_*o*_)/ε,^[^ ^29^, ^30^ ^]^ where Δ*R* is the resistance variation, *R*_*o*_ is the initial resistance, and ε is the applied strain. Figure 2d shows an increase in the resistance variation with *GF* = 24.28 for strain less than 2%, and *GF* = 12.23 for higher strains.

### 2.3 Sensing Mechanism and Analytical Model

**Figure 3:**
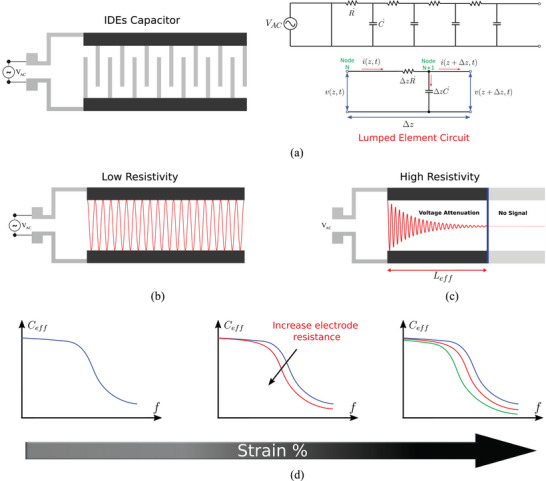
Signal propagation in IDEs capacitive sensors a) Schematic of IDEs capacitor structure with transmission line model as a network of R–C chains with an equivalent lumped element circuit b) Signal propagation in IDEs with low resistivity electrodes c) Signal propagation in IDEs with high‐resistivity electrodes d) Graphical illustration of the variation in capacitance with frequency due to transmission line activation at different strain levels.

The capacitance variation that causes a remarkable shift in the resonant frequency cannot be achieved in classical parallel capacitive sensors, particularly at low strain levels. To address this issue and enhance the sensitivity, we integrated the piezoresistive behavior with a planar IDEs capacitor to enable a variable resistance transmission line and move away from geometrically driven sensing to resistivity and frequency‐based interrogation sensing mechanism. Capacitive sensors with high resistive electrodes can function as a transmission line and be represented as a resistor‐capacitor (R‐C) chain.^[^ ^31^, ^32^, ^33^, ^34^, ^35^, ^36^ ^]^ The IDEs capacitor has the same working principle as the parallel plate capacitor because the finger‐like periodic pattern of parallel in‐line electrodes was used to build up the capacitance associated with the electric field penetrating the material.^[^ ^37^ ^]^ Therefore, transmission line theory was used here to model and analyze the IDE capacitor with piezoresistive electrodes and represent it as an infinite series of RC chains as shown in **Figure** **3**a.

The transmission line theory was used to analyze the equivalent lumped element model of IDEs sensor and describe its internal response. By solving the telegrapher's equations, the voltage equation of the electric signal can be expressed as Equation (2). A complete analytical model is described in the supporting information (Section 3).

(2) $$V\left(z\right)=V_{o}e^{-\alpha z}cos\left(2\pi ft-\beta z\right)$$

where z is the propagation direction along the length of the sensor, *V*_*o*_ is the magnitude of the alternative input voltage, α is the attenuation factor, *f* is the frequency of the interrogation signal and β is the phase constant.

Figure 3b,c illustrates how the voltage signal changes along the transmission line at two different electrode resistances with a constant frequency. For graphical understanding, short‐wavelength oscillations are represented here, but let us note that in the real application, the wavelength is much larger than the scale of the sensor. In cases where electrodes with low resistivity and/or low frequency are used, the voltage signal travels throughout the length of the sensor and charges each capacitor equally along the path (Figure 3b). In cases where high‐resistive electrodes are used (Figure 3c), the signal gradually attenuates until it completely vanishes and can no longer charge the capacitors. This results in a virtual or effective length at which the signal is fully attenuated as expressed in Equation (3), and the new capacitance is calculated based on the new effective length as expressed in Equation (4).^[^ ^35^, ^36^ ^]^

(3) $$L_{eff}=-\frac{\ln\left(\frac{V_{min}}{V_{o}}\right)}{\sqrt{\pi f_{s}C^{\prime }R^{\prime }}}$$

(4) $$C_{eff}=C_{o}\left(1+\varepsilon \right)\left[-\frac{ln\left(\frac{V_{min}}{V_{o}}\right)}{\sqrt{2\pi f_{s}C_{o}\left(1+\varepsilon \right)R\left(\varepsilon \right)}}\right]$$

where *C*′ and *R*′ are the unit length capacitance and resistance respectively, *V*_*min*_ is the voltage magnitude at distance (*L*_*eff*_), *C*_*o*_ is the initial IDEs capacitance, and ε is the applied strain.

Thus, the penetration depth of the interrogation signal depends on the resistivity of the electrode and interrogation frequency. An increase in the electrode resistivity increases the signal attenuation, and reduces the penetration depth along the sensor length, thereby considerably reducing the effective capacitance. In contrast, the penetration depth of the signal can be modulated by changing the frequency because at a certain resistivity level, increasing the frequency has the same effect as resistivity on decreasing the penetration depth of the interrogation signal (Figure 3d).

### 2.4 Performance of the Interdigitated Electrodes (IDEs) Capacitor

**Figure 4:**
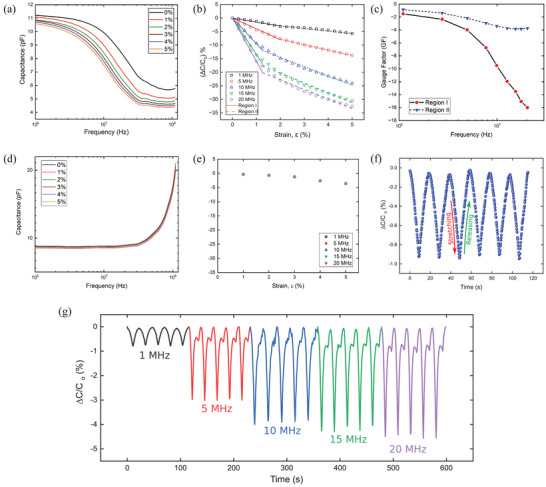
a) Capacitance variation of the IDEs capacitor with frequency sweep at different strain levels. b) The relative capacitance variation of IDEs capacitor with piezoresistive electrodes versus strain. c) Relationship between interrogation frequency and sensitivity of IDEs with piezoresistive electrodes d) Capacitance variation of classical IDEs with a highly conductive electrode subjected to tensile strain at different frequencies. e) Relative change in the capacitance of IDEs with highly conductive electrodes. f) Relative variation in capacitance during stretching and releasing at 1 MHz. g) Effect of the signal frequency on the measured capacitance during dynamic measurements.

The effect of the transmission line on the measured capacitance was determined by fabricating two configurations of IDEs capacitors and subjecting them to strain. The capacitance was measured at different strains using an impedance analyzer with a frequency range from 40 Hz to 110 MHz. First, 1 × 2 cm^2^ IDEs with piezoresistive electrodes were stretched until they reached 5% strain. Indeed, for many structural applications, we are targeting to monitor strain in a low range to detect damage and failure indicators at an early stage. The capacitance was measured as shown in **Figure** **4**a, which varied with frequency and strain. As discussed in Section 2.3, the penetration depth of the interrogation signal is dependent on frequency and resistance, and increasing both or either of these parameters will enhance the capacitance variation due to the signal attenuation along the sensor length. This is very clear in Figure 4a, whereas for each strain level, we can see a drastic reduction in the capacitance by increasing the frequency from 1 to 110 MHZ. At a certain frequency, the capacitance decreased with strain (Figure 4b) due to a change in the resistance of the piezoresistive electrodes as presented in the piezoresistivity curve in Figure 2d.

Figure 4b shows a high relative variation of the effective capacitance at low strain levels to achieve 5.7% at 1 MHz and 32.6 % at 20 MHz at 5% strain. Figure 4b can be divided into two linear regimes; Regions I and II are represented by solid and dashed lines respectively for the detection of very low and high strain values, respectively. The sensitivity of IDEs capacitive sensor over frequency in these two regions is shown in Figure 4c. The sensitivity (GF) in Region I, increases from 1.46 to 16.8 at higher frequencies of 1 and 20 MHz, respectively. In Region II, the slope of the capacitance variation (Figure 4b) varies, which decreases the sensor sensitivity at different frequencies (Figure 4c). These results show the capability of the capacitive sensor to detect minimal strains in different structures with high sensitivity, in addition to controlling the GF by modulating the signal frequency. The GF for the capacitive sensor was calculated using Equation (5):

(5) $$GF=\frac{\frac{\Delta C}{C_{o}}}{\varepsilon }$$

where Δ*C* is the relative change in capacitance, and *C*_*o*_ is the initial capacitance.

Moreover, another IDEs capacitive sensor with the same dimensions was fabricated using conductive electrodes. It was analyzed for characterizing the capacitance variation versus frequency at different strain levels as shown in Figure 4d. Almost no variation in the capacitance was observed for this sensor over the frequency band up to 20 MHz due to the low initial resistance of the conductive electrodes which maintains its low resistance with strain (Figure 2c). However, after 20 MHz, the capacitance increased because of approaching the self‐resonant frequency (SRF), this increased the effect of the parasitic elements.^[^ ^38^, ^39^ ^]^ As shown in Figure 4e, the variation in capacitance versus strain is very small, and this is due to the small variation in electrode resistance. Moreover, there is no variation in capacitance due to stretching, regardless of the signal frequency, even at higher frequencies. Therefore, for IDEs capacitors with purely conductive ink, the effect of resistance variation and frequency modulation were insignificant, compared to those with piezoresistive electrodes.

A cyclic test was performed on the IDEs capacitor with piezoresistive electrodes to characterize the dynamic response of the sensor by applying cyclic strain ranges from 0% to 2% with a strain rate of 5 mm per min. The sensor detected the variation in capacitance during stretching and releasing as presented in Figure 4f in which the capacitance was measured at a frequency of 1 MHz. To investigate the effect of interrogation frequency on the measured capacitance during dynamic measurements, we varied the interrogation frequency varied during the cyclic test. As the signal frequency increased, the amplitude of relative changes in capacitance also increased, implying higher sensitivity as shown in Figure 4g. These results are highly significant as they demonstrate the ability of the sensor to control its sensitivity by adjusting the frequency. This enables the precise measurement of specific strains while effectively canceling out a considerable amount of noise that may originate from environmental sources. Such enhancements are crucial for improving the accuracy and reliability of the fabricated sensor performance in diverse conditions.

**Figure 5:**
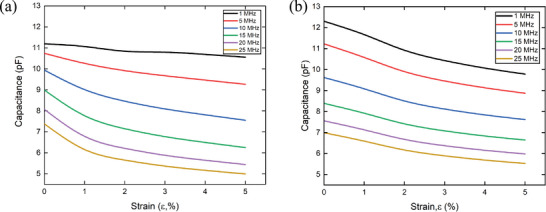
Validation of the analytical for the IDEs Capacitive Sensor a) Analytical results, and b) Experimental results.

The experimental results of the IDEs capacitor testing (**Figure** **5**a) were used to validate the analytical model developed based on the transmission line theory (refer to Section S3, Supporting Information for the full analytical model). Based on the piezoresistivity response of the IDEs electrodes as reported in Figure 2d, and assuming that *V*_*min*_ = 0.1*V*_*o*_, the effective capacitance was calculated analytically using Equation 4 at different operating frequencies as shown in Figure 5a. A comparison of experimental and analytical results (Figure 5a,b) shows a good correlation. The difference between analytical and experimental results can be attributed to the initial assumption in deriving the transmission line model as discussed in the Supporting Information, in addition to neglecting the effect of the parasitic elements that exist in the sensor.

### 2.5 Strain Detection Using Wireless Printed LC Sensor

**Figure 6:**
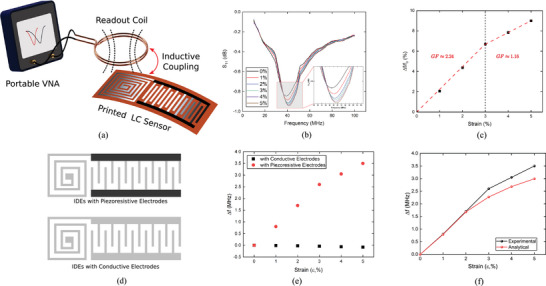
a) Schematic of the wireless strain detection via electromagnetic coupling using an external coil connected to the vector network analyzer. b) Shifting in the resonant frequency of the LC sensor due to stretching by measuring the reflection parameter (*S*_11_). c) Relative change in the resonant frequency of the wireless LC sensor versus strain with the sensitivity of the sensor in wireless strain detection. d) Two tested configurations of the LC‐printed sensors. e) Comparison of the change in resonant frequency of LC sensor using conductive and piezoresistive electrodes. f) Comparison of the analytical and experimental results in predicting the resonance frequency shifting due to stretching.

The primary aim of this study was to develop a sensor using a low‐cost and simple fabrication method for collecting strain data wirelessly. To collect data remotely, we rely on RF technology using an external readout coil connected to a Vector Network Analyzer (VNA). This coil transmits a signal with a frequency sweep range, including the resonant frequency of the LC circuit. Then, the signal was received through the planar coil printed on the sensor via resonant inductive coupling, as illustrated in **Figure** **6**a to resonate with the LC sensor on its resonant frequency. The reflection coefficient (*S*_11_) was measured, indicating that the sensor initially resonated at *f* = 38.9 MHz. As the capacitance changed during stretching, the resonant frequency shifted considerably as shown in Figure 6b. Figure 6c, shows that the sensor can detect strain variations with a GF of 2.24 for strains less than 5%. For higher strains, a GF of 1.16 was achieved.

To investigate the effect of piezoresistive electrodes on wireless detection Two printed sensors were fabricated: one with piezoresistive electrodes and the other with only conductive electrodes (Figure 6d). The results showed that the first sensor with piezoresistive electrodes showed much higher sensitivity with a relative change in the resonant frequency up to 9% at 5% strain (Figure 6e). In contrast, the sensor with only conductive ink almost shows no variation in the resonant frequency at low strain levels. In Figure 6.f, the developed analytical model was used to predict the response of the wireless sensor by considering not only the variation in capacitance due to the transmission line effect. As shown in Figure 6f, the experimental and analytical results showed a good correlation, particularly at low strain values. This confirms the accuracy of the analytical model in predicting the sensor response by considering the transmission line effect.

**Figure 7:**
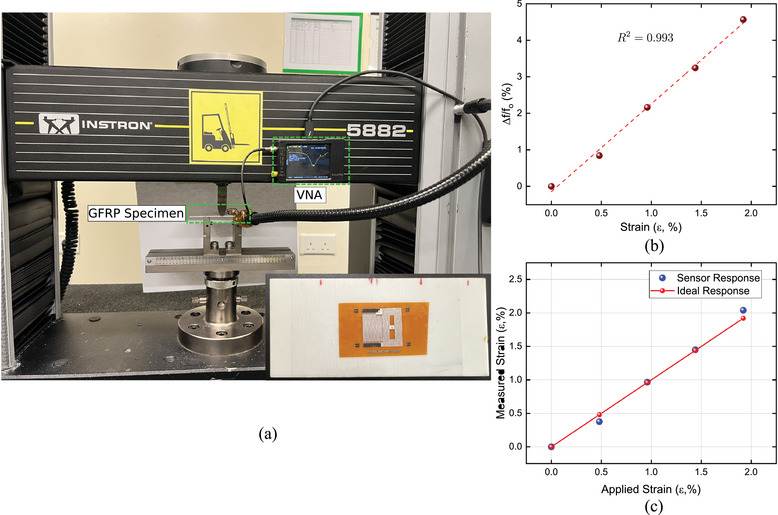
a) Bending test setup of GFRP specimen with integrated LC printed sensor. b) Relative variation of resonant frequency due to the applied strain. c) Comparison between the applied and measured strain.

The high flexibility and sensitivity of the developed printed sensor enable it to be integrated with different composite structures such as glass fiber reinforced polymer (GFRP) or carbon fiber reinforced polymer (CFRP) either by attaching to the surface externally or by internally embedding between the laminates. The sensor performance in detecting the strain in GFRP was demonstrated via a bending test. During the test, the sensor was attached to the bottom surface of GFRP (**Figure** **7**a). Three‐point bending was performed and the variation of resonant frequency was recorded using NanoVNA equipped with an external readout coil fixed on the top surface of the GFRP specimen.

The applied displacement during the bending test caused a stretching of the attached sensor. This deformation activated the transmission line model and reduced the effective capacitance which resulted in a shift in the resonant frequency. Figure 7b shows the relative variation of the resonant frequency during the bending test with a perfect linearity. This confirms that the developed sensor can detect low‐strain values remotely. To calibrate the sensor response, applied strain versus measured strain was plotted as presented in Figure 7c. Whereas the applied flexural strain can be calculated as (ε = 6*dh*/*L* ^2^), where *d* is the maximum deflection of the mid‐point of the specimen, *h* is the thickness of the test sample and *L* is the distance between the two supports. The measured strain was calculated as $\varepsilon =\frac{\left(\Delta f/f_{o}\right)}{GF}$ using *GF* = 2.24 as deduced from Figure 6c that characterizes the wireless sensor. The strain values measured by the wireless sensors showed a good match with the applied strain (Figure 7c), indicating that the sensor response matched the ideal response (meaning the measured strain matched exactly the applied strain).

## 3 Conclusion

Herein, a flexible and wireless strain sensor was fabricated by screen printing. The sensor structure comprised an LC resonator with a distinct resonance frequency that used capacitance as a sensing unit. The variation in capacitance due to stretching caused a shift in the resonance frequency. A piezoresistive material was used to fabricate a piezocapacitive sensor for activating the transmission line. The sensor detected minimal strain variation with high sensitivity corresponding to the changes in the penetration depth of the interrogation signal. Two inks (conductive and piezoresistive) were printed on a flexible PI substrate using a screen printing technique, and electromechanical tests were performed to characterize the sensor performance. Data were collected wirelessly via electromagnetic coupling between an external coil connected to VNA and the receiver coil in the sensor. The reflected signal was recorded and analyzed at different strains. As a capacitive sensor, the IDEs capacitor was tested and characterized to demonstrate its feasibility in enabling transmission line using piezoresistive ink. It achieved optimal sensitivity with GF ≈16 at 20 MHz. The sensor showed a significant shift in the resonance frequency during stretching with GF ≈2.2. To demonstrate the potential of the sensor in structural health monitoring (SHM) applications of composite material the sensor was used to measure the applied strain on the GFRP specimen during the bending test, and it was able to detect strain at low values (less than 2%).

## 4 Experimental Section

### Fabrication and Characterization

Two mesh screen masks were designed to print the IDEs capacitor in two layers of conductive and piezoresistive inks. First, a silver‐based conductive and stretchable ink (Creative Materials Inc., USA) was used to print the conductive layer of the sensor coil and IDEs electrodes on a 50µm PI film followed by 10 min of curing at 175 °C in the oven. After curing, the conductive layer was aligned through the printed alignment marks, and a second screen was used to print piezoresistive electrodes using carbon‐based ink (DycoTec Materials Ltd., UK), followed by curing at 135 °C for 20 min. For electromechanical testing, a 30 AWG flexible silicon electric wire was attached to the IDEs capacitive sensor by a silver conductive epoxy adhesive (M.G. Chemicals Ltd., Canada) and cured for 10 min at 65 °C. The quality of the printed structures was investigated by imaging them under a stereo microscope (Zeiss, Germany). Additionally, 3D thickness measurements of the printed electrodes were performed using an optical profiler (Zygo Corporation, USA).

### Electromechanical Testing

To quantify the electromechanical performance of the printed structures, a static tensile test was performed using a 5994 Instron universal machine by applying a uniaxial strain at a strain rate of 1 mm per min. The instantaneous electrical resistance was simultaneously recorded using a KEYSIGHT 34461A digital multimeter. A KEYSIGHT impedance analyzer E4294 with frequency ranges from 40 Hz to 110 MHz was used to measure the capacitance during the mechanical testing of the capacitive IDEs sensor. To characterize the dynamic response of the printed IDEs sensor, a cyclic test was performed using a 5994 Instron universal machine at a strain rate of 5 mm per min. Then, the capacitance was measured in real‐time using a KEYSIGHT impedance analyzer (E4294) connected to a LabView program to record the real‐time data.

### Wireless Strain Detection

To measure strain using a wireless system, an external read‐out coil was connected to a vector network analyzer (Agilent N5225A). An electromagnetic (EM) signal with a frequency sweep, including the resonant frequency, was transmitted through an EM coupling formed between the receiver and transmitter coils to resonate the sensor. The reflected signal was acquired and represented by the reflection coefficient (*S*_11_) which expresses the ratio between the reflected signal power to the incident power.

### GFRP Specimen Fabrication and Bending Test

An impact‐modified polypropylene copolymer (IPP) reinforced with continuous E‐glass fibers was used to fabricate the GFRP specimens. Eight 0.25 mm thick layers of unidirectional laminates were stacked in a customized mold and consolidated in a hot press at a temperature of 280 °C and pressure of 7.5 bar. A universal testing machine (Instron E3000) was used to conduct a three‐point bending test for the fabricated GFRP specimens. The printed sensor was attached to the bottom surface of the specimen using a Skotch weld PR40 (3M, US). For measuring the reflection coefficient, NanoVNA equipped with an external readout coil was used to send an EM signal and receive the reflected signal. The coil was fixed on the top surface of the GFRP sample.

## Conflict of Interest

The authors declare no conflict of interest.

## Author Contributions

H.M., H.N., and G.L. conceived and designed the research. H.M. fabricated the sensors and performed the experiments. H.M. and T.M. performed tests and analyzed the data. H.M., H.N., and T.M. wrote the manuscript. H.N. and G.L. supervised the data analysis. G.L. and S.A. supervised the research. All authors were involved in the discussion and finalization of the manuscripts.

## Supporting information

Supporting Information

Supplemental Video S1

Supplemental Video S2

## Data Availability

The data that support the findings of this study are available from the corresponding author upon reasonable request.
