# Supplementary material for: A Fully Printable Strain Sensor Enabling Highly‐Sensitive Wireless Near‐Field Interrogation
Source: Adv Sci (Weinh). 2025 Jan 21;12(9):2411346. doi: 10.1002/advs.202411346 (PMC11884597; doi:10.1002/advs.202411346)
Supplement: Supplementary file 1 — Supporting Information [file ADVS-12-2411346-s002.pdf]

ADVANCED SCIENCE
Open Access

Supporting Information

for *Adv. Sci.*, DOI 10.1002/advs.202411346

A Fully Printable Strain Sensor Enabling Highly-Sensitive Wireless Near-Field Interrogation

*Hassan A. Mahmoud, Hussein Nesser, Tarek M. Mostafa, Shehab Ahmed and G. Lubineau**

Supporting Information: A Fully Printable Strain Sensor Enabling Highly-Sensitive Wireless Near-Field Interrogation

Hassan A. Mahmoud[1,2], Hussein Nesser[1,2], Tarek M. Mostafa[3], Shehab Ahmed[3], G. Lubineau[1,2*]

[1] *Mechanical Engineering Program, Physical Science and Engineering Division, King Abdullah University of Science and Technology (KAUST), Thuwal 23955-6900, Kingdom of Saudi Arabia*

[2] *Mechanics of Composites For Energy and Mobility Lab, King Abdullah University of Science and Technology (KAUST)*

[3] *CEMSE Division, King Abdullah University of Science and Technology, Thuwal 23955, Saudi Arabia*

[*] Corresponding author. Tel: +966 (12) 808-2983
Email address: gilles.lubineau@kaust.edu.sa (G. Lubineau[1,2])

1. Sensor Design and Calculation

The printed sensor comprises a planar spiral coil connected to an interdigitated electrodes (IDEs) capacitor that forms an LC resonant circuit as shown in Figure S1.a. The inductance (L) of a planar spiral coil can be calculated using the Modified Wheeler Formula [1] as expressed in Equation S1

$$L_S = K_1 \mu_o \frac{n^2 d_{avg}}{1 + K_2 \rho} \tag{S1}$$

where K_1 and K_2 are layout-dependent coefficients [1], μ_o is magnetic permittivity in free space, ρ is the fill ratio (how hollow is the inductor), $\rho = \frac{d_{out} - d_{in}}{d_{out} + d_{in}}$, n is the number of turns, d_{avg} is the average coil diameter, $d_{avg} = \frac{d_{out} + d_{in}}{2}$.

The IDEs capacitor comprises a special type of electrode designed in a finger/comb shape with a periodic interlocking pattern and deposited on a substrate as illustrated in Figure S1.b. The capacitance of the IDEs can be deduced from the capacitance of the unit cell per unit length (C_{UC}) (Figure S1.c) which is written in Equation S2.

$$C_{UC} = C_1 + C_2 + C_3 \tag{S2}$$

The capacitance of the unit cell (C_{UC}) is composed of three partial capacitances C_1, C_2, and C_3 correspond to the permittivity ε_1, ε_2, and ε_3. The upper and lower semi-planes of a unit cell with vanishing electrode thickness ($h = 0$) are described by the capacitance C_1 and C_3. They are calculated by conformal mapping of the two semi-planes with permittivity ε_1 (usually the permittivity of the encapsulant, or of air for the non-encapsulated sensors) and ε_3 (usually the permittivity of the substrate, or of air for self-standing sensors) into two plate capacitors, using a complete elliptical integral of the first kind ($K[x]$) as a transformation [2, 3]:

$$C_1 + C_3 = \varepsilon_o \frac{(\varepsilon_1 + \varepsilon_3)}{2} \frac{K\left[\sqrt{1 - \left(\frac{a}{b}\right)^2}\right]}{K\left[\frac{a}{b}\right]} \tag{S3}$$

where $\varepsilon_o = 8.85.10^{-12} F/m$ is the relative permittivity of the free space and K[x] is the

elliptical integral of the first kind.

For the non-vanishing thickness (h) of the electrodes, the volume between the electrodes is treated like a plate capacitor with permittivity ε_2, and the capacitance (C_2 per unit length can be written as:

$$C_2 = \varepsilon_o \varepsilon_2 \left(\frac{h}{a} \right) \tag{S4}$$

By combining Equations S3 and S4, (C_{UC}) can be written as:

$$C_{UC} = C_1 + C_2 + C_3 = \varepsilon_o \frac{(\varepsilon_1 + \varepsilon_3)}{2} \frac{K\left[\sqrt{1 - \left(\frac{a}{b}\right)^2}\right]}{K\left[\frac{a}{b}\right]} + \varepsilon_o \varepsilon_2 \left(\frac{h}{a} \right) \tag{S5}$$

Then, total initial capacitance of the IDE can be expressed as:

$$C_S = l(N-1)C_{UC} \tag{S6}$$

$$C_S = l(N-1) \left[\varepsilon_o \frac{(\varepsilon_1 + \varepsilon_3)}{2} \frac{K\left[\sqrt{1 - \left(\frac{a}{b}\right)^2}\right]}{K\left[\frac{a}{b}\right]} + \varepsilon_o \varepsilon_2 \left(\frac{h}{a} \right) \right] \tag{S7}$$

where C_S is the total initial capacitance of the IDEs capacitor, N is the number of unit cells, l is the length of the electrode finger

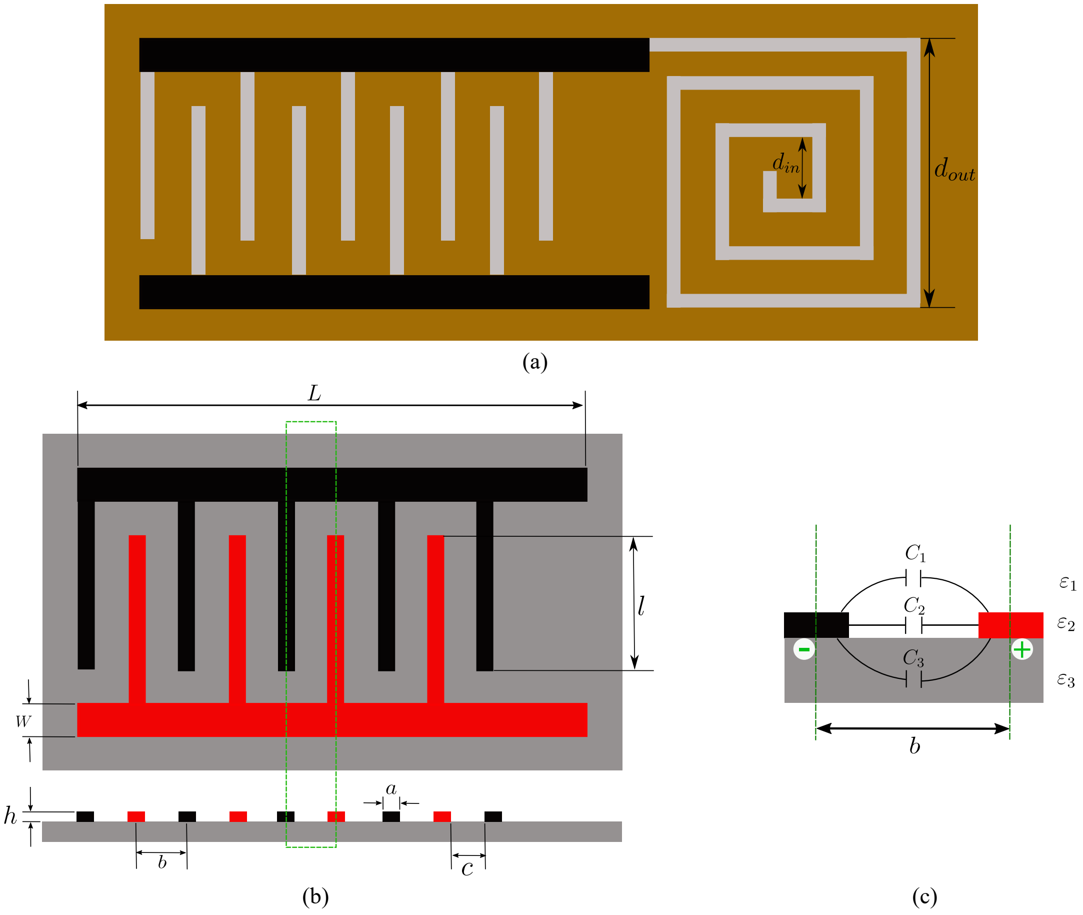

Figure S1: (a) Structure of LC printed sensor consisting of interdigitated electrode (IDEs) capacitor connected to a planar spiral coil. (b) Schematic of IDEs capacitive sensor with geometrical notations (c) IDEs unit cell with equivalent capacitance

Knowing the initial inductance (L_S) and capacitance (C_S), the initial resonance frequency (f_r) of the LC resonant circuit can be calculated as

$$f_r = \frac{1}{2\pi\sqrt{L_S C_S}} \tag{S8}$$

A key aspect of our technology is to measure the attenuation in the interrogation signal with a change in the resonance frequency. It is then essential that the dissipation is significant at a frequency close to the interrogation frequency. f_r and the attenuation response should be consistent with a careful sensor design. Section 3 highlights the key equations that describe the dissipation of the signal.

2. LC sensor Fabrication

After printing the inductor and capacitor of the LC resonant circuit, a jumper bridge needs to be created to close the LC circuit. To do this, we created two vias as illustrated in Figure S2.a and used conductive silver-based ink (Creative Materials Inc., USA) to print a jumper on the backside of the substrate between the two vias as shown in Figure S2.b.

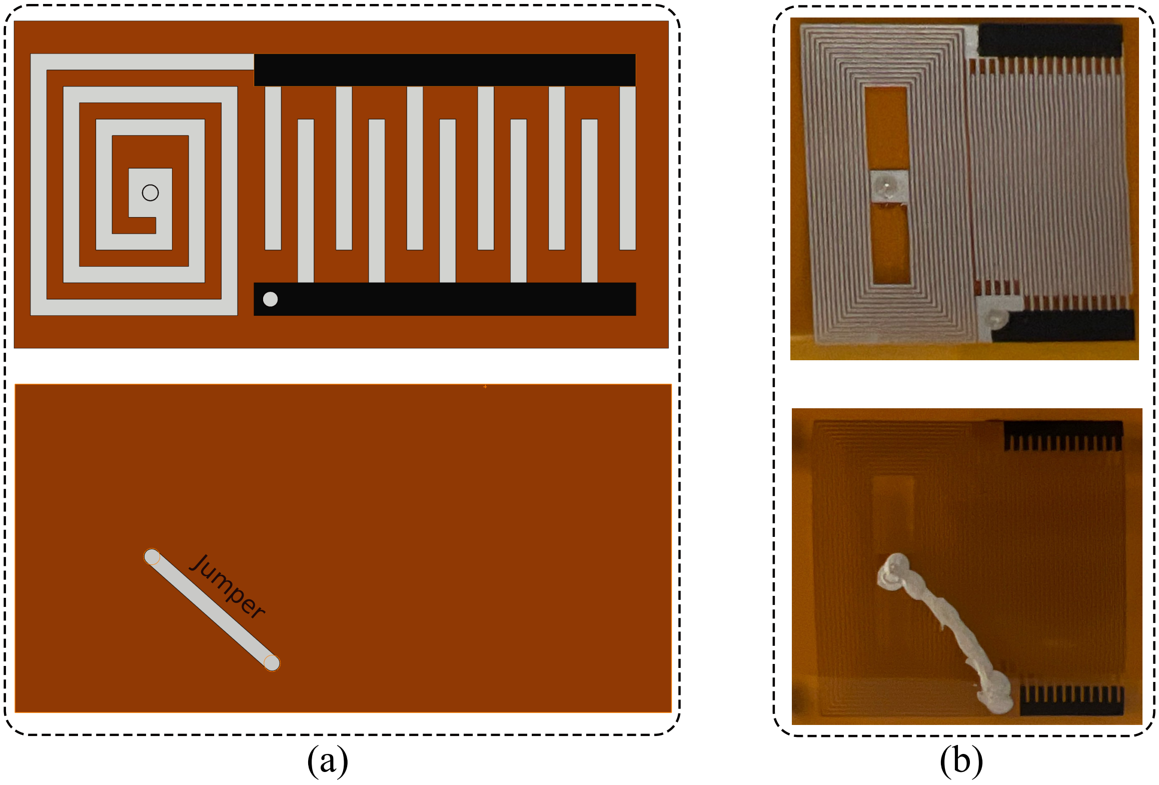

Figure S2: (a) A Schematic illustration of the circuit jumper created to close the LC circuit (b) An optical image of the created jumper on the backside of the substrate

3. Transmission Line Analytical Model of IDEs Sensor

In this section, we recall the key equations that describe the dissipation of the signal along the IDE capacitor.

Transmission Line Equations

The interdigitated electrodes (IDEs) sensor operates on the same principle as two parallel plate capacitors. This finger-like periodic pattern of parallel in-line electrodes builds up the capacitance associated with the electric fields penetrating the material. Hence, the transmission line model of the parallel plate capacitor is used to analyze the IDEs sensor [4] as illustrated in Figure S3.a. The IDEs capacitor can be represented as an infinite series of RLCG circuits (resistance, inductance, capacitance, and conductance), as shown in Figure S3.a.

We divided the transmission line into differential sections, each of length (Δz). Each section was represented by an equivalent circuit of lumped elements as shown in Figure S3.b. In this model, we simplified the lumped circuit by neglecting the self-inductance (L) in the IDEs and the conductance of the dielectric layer (G).

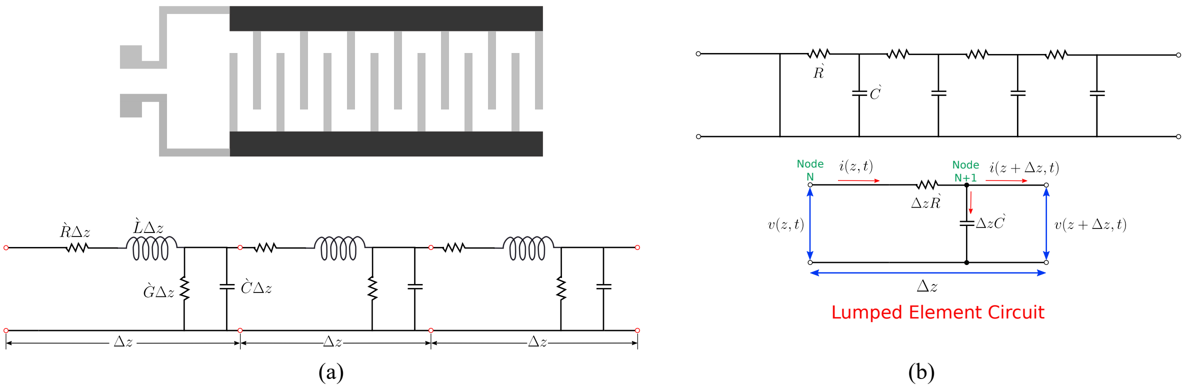

Figure S3: (a) Equivalent transmission line model of IDEs capacitor as RLCG chain. (b) Equivalent lumped element of circuit used in analyzing IDEs capacitive sensor.

Transmission line theory was used to analyze the lumped element and describe the voltage across the line as a function of time (t) and spatial position (z). The lumped-element circuit model (Figure S3.b) was used to consider a deferential length Δz where $v(z,t)$ and $i(z,t)$

denote the instantaneous voltage and current at the left end of the differential section (Node N), and similarly $v(z+\Delta z, t)$ and $i(z+\Delta z, t)$ denote the same quantities at the right end (Node N+1). By Applying Kirchhoff's current and voltage laws to the lumped element circuit model, we can obtain the equations of the transmission line in the time domain (telegrapher's equations) as follows:

By Applying Kirchhoff's Voltage Law (KVL)

$$v(z,t) - R^{'}\Delta z\ i(z,t) - v(z+\Delta z, t) = 0 \tag{S9}$$

Upon dividing all terms by Δz and rearranging terms we obtain

$$-\left[\frac{v(z+\Delta z,t) - v(z,t)}{\Delta z}\right] = R^{'}\ i(z,t) \tag{S10}$$

In the limit as $\Delta z \to 0$. Equation S10 becomes a differential equation:

$$\frac{-\partial v(z,t)}{\partial z} = R^{'} i(z,t) \tag{S11}$$

By Applying Kirchhoff's Current Law (KCL) at node (N +1)

$$i(z,t) - C^{'}\Delta z \frac{\partial v(z+\Delta z)}{\partial t} - i(z+\Delta z, t) = 0 \tag{S12}$$

Upon dividing all terms by Δz and rearranging the terms, we obtain

$$-\left[\frac{i(z+\Delta z,t) - i(z,t)}{\Delta z}\right] = C^{'}\frac{\partial v(z+\Delta z)}{\partial t} \tag{S13}$$

In the limit as $\Delta z \to 0$. Equation S13 becomes a differential equation:

$$\frac{-\partial i(z,t)}{\partial z} = C^{'}\frac{\partial v(z,t)}{\partial t} \tag{S14}$$

The first-order differential equations are given by Equations S11 and S14, which describe the voltage and current relationships in a transmission line and are known as telegrapher equations. For the sinusoidal steady-state condition, with a cosine-based phasor, we can

assume that both voltage and current are time-harmonic. Hence, Equations S11 and S14 can be written in the phasor domain as:

$$-\frac{dV(z)}{dz} = R^{'}I(z) \tag{S15}$$

$$-\frac{dI(z)}{dz} = j\omega C^{'}V(z) \tag{S16}$$

Wave Propagation on a Transmission Line

By combining the first-order equations S15 and S16 to obtain a second-order voltage wave equation as:

$$\frac{d^2V(z)}{dz^2} - \gamma^2 V(z) = 0 \tag{S17}$$

where γ is a complex propagation constant, $\gamma = \sqrt{j\omega C' R'}$, and ω is the angular frequency of the input signal.

The solution of the traveling wave equation S17 can be expressed as a complex exponential in phasor form as:

$$V(z) = V_o^+ e^{-\gamma z} + V_o^- e^{+\gamma z} \tag{S18}$$

where V_o is the magnitude of the input voltage, V_o^-, and V_o^+ represents the magnitude of voltage of waves that travel along the transmission line in forward and backward directions. The terms include $e^{-\gamma z}$, and $e^{+\gamma z}$ refer to the propagation in $+z$ direction (forward propagation) and $-z$ direction (backward propagation), respectively.

Recall the equations given by Eq. (S11) and (S12) and considering $Z = L$, there is no $i(z + \Delta z.t)$ to obtain one equation in the voltage form.

$$\frac{\partial v(z,t)}{\partial t} + R^{'} C^{'} \frac{\partial v(z,t)}{\partial t} = 0 \tag{S19}$$

Using the phasor form of the voltage $V(z,t) = V(z)e^{j\omega t}$. Equation S19 can be written as:

$$\begin{gathered} \frac{dV(z)}{dz} + R^{'} C^{'} j\omega V(z) = 0 \\ \frac{dV(z)}{dz} + \gamma^2 V(z) = 0 \end{gathered} \tag{S20}$$

Substitute using Equation S18 in Equation S20 and simplify the equation to solve for V_o^+

$$V_o^+ = \frac{V_o}{\left[e^{-2\gamma z}\dfrac{1-\gamma}{1+\gamma}+1\right]} \tag{S21}$$

where L is the sensor length. and the propagation constant γ is a complex number depending on the capacitance and electrode resistance per unit length, $C^{'}$ and $R^{'}$ respectively.

Consider that the voltage of the input signal is vanishing at the highly resistive electrode and/or at high frequency and assume that $e^{-2\gamma z} \approx 0$, and $\gamma >>> \frac{1}{2L}$, hence $V_o^- = 0$ and $V_o^+ = V_o$. Therefore, Equation S18 can be simplified to:

$$V(z) = V_o e^{-\gamma z} \tag{S22}$$

Rewrite Equation S22 in the time domain as follows:

$$V(z,t) = V_o e^{-\alpha z} cos(\omega t - \beta z) \tag{S23}$$

where $\alpha = \sqrt{\pi f R^{'} C^{'}}$ is the attenuation factor for the traveling wave, $cos(\omega t - \beta z)$ is the oscillation factor of the sinusoidal signal with β as a phase constant, $\beta = Im(\gamma) = \frac{2\pi}{\lambda}$, λ is the wave length, ω is the angular frequency, $\omega = 2\pi f$, V_o is the magnitude of the input voltage.

Effective Length Calculation

Under strain (high electrode resistance), the voltage wave amplitude decreases gradually until fully attenuated. The distance at which the signal is fully attenuated is called the effective length (L_{eff}) and the voltage will reach ($V_o = V_{min}$) [5, 6]. Recalling Equation S22 and considering the real part only, it can be written as:

$$V_{min} = V_o e^{-\alpha L_{eff}} \tag{S24}$$

Hence, the effective length can be written as follows:

$$L_{eff} = -\frac{ln\left(\frac{V_{min}}{V_o}\right)}{\sqrt{\pi f C' R'}} \tag{S25}$$

where $R' = \frac{R}{L}$, since R is the total resistance of the electrodes and $C' = \frac{C}{L} = \frac{C_s(1+\varepsilon)}{L}$, since C_s is the initial capacitance of the sensor.

Recall Equation S6 that calculates the initial capacitance of IDEs and considers the number of unit cells as ($N = \frac{L}{b}$), and rewrite the equation as:

$$C_s = C_{UC} l \left(\frac{L}{b} - 1\right) \tag{S26}$$

Equation S26 can be simplified by neglecting 1 for a high number of unit cells and rewritten as:

$$C_s = C_{UC} \left(\frac{l}{b}\right) L \tag{S27}$$

When subjecting the IDEs capacitive sensor to strain, the capacitance can be calculated as:

$$C = C_s(1+\varepsilon) = C_{UC} \left(\frac{l}{b}\right) L(1+\varepsilon) \tag{S28}$$

In the case of a transmission line, the effective capacitance can be written as:

$$C_{eff} = C_{UC} \left(\frac{l}{b}\right) (1+\varepsilon) L_{eff} \tag{S29}$$

Using the effective length calculated in Equation S25, the effective capacitance can be expressed as:

$$C_{eff} = C_s(1+\varepsilon)\left[-\frac{\ln\left(\dfrac{V_{min}}{V_o}\right)}{\sqrt{2\pi f C_s(1+\varepsilon)R(\varepsilon)}}\right] \tag{S30}$$

Where C_{eff} is the effective capacitance calculated based on the length at which the signal is fully attenuated (effective length, L_{eff}).

References

[1] S. S. Mohan, M. del Mar Hershenson, S. P. Boyd, T. H. Lee, Simple accurate expressions for planar spiral inductances, IEEE Journal of solid-state circuits 34 (10) (1999) 1419–1424.

[2] H.-E. Endres, S. Drost, Optimization of the geometry of gas-sensitive interdigital capacitors, Sensors and Actuators B: Chemical 4 (1-2) (1991) 95–98.

[3] A. Abu-Abed, R. Lindquist, Capacitive interdigital sensor with inhomogeneous nematic liquid crystal film, Progress In Electromagnetics Research B 7 (2008) 75–87.

[4] A. V. Mamishev, K. Sundara-Rajan, F. Yang, Y. Du, M. Zahn, Interdigital sensors and transducers, Proceedings of the IEEE 92 (5) (2004) 808–845.

[5] H. Nesser, G. Lubineau, Achieving super sensitivity in capacitive strain sensing by electrode fragmentation, ACS Applied Materials & Interfaces 13 (30) (2021) 36062–36070.

[6] H. Nesser, G. Lubineau, Minimizing the wiring in distributed strain sensing using a capacitive sensor sheet with variable-resistance electrodes, Scientific reports 12 (1) (2022) 13950.
